# Genomic medicine for heart failure prediction in patients with atrial fibrillation

**DOI:** 10.1093/europace/euaf113

**Published:** 2025-06-06

**Authors:** Leonoor F J M Wijdeveld, Sean J Jurgens

**Affiliations:** Department of Physiology, Amsterdam Cardiovascular Sciences, Heart Failure & Arrhythmias, Amsterdam UMC Location Vrije Universiteit, Amsterdam, De Boelelaan 1117, 1081 HV, the Netherlands; Cardiovascular Disease Initiative, Broad Institute of MIT and Harvard, Cambridge, 415 Main Street Cambridge, MA 02142, USA; Cardiovascular Disease Initiative, Broad Institute of MIT and Harvard, Cambridge, 415 Main Street Cambridge, MA 02142, USA; Department of Experimental Cardiology, Amsterdam Cardiovascular Sciences, Heart Failure & Arrhythmias, Amsterdam UMC Location University of Amsterdam, Meibergdreef 9, Amsterdam 1105AZ, the Netherlands

## Abstract

**Graphical Abstract:**
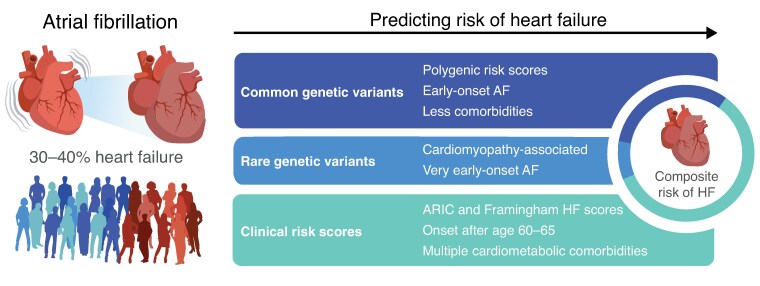

**This editorial refers to ‘Polygenic risk-based prediction of heart failure in young patients with atrial fibrillation: an analysis from the UK Biobank’, by Hyo-Jeong Ahn *et al.*, https://doi.org/10.1093/europace/euaf104.**

Atrial fibrillation (AF) is a highly prevalent disorder of the cardiac rhythm. While there is a strong clinical emphasis on stroke risk and stroke prevention, AF also represents a major risk factor for heart failure (HF). In fact, 30–40% of AF patients will develop HF during their lifetime, while only 10–20% will experience an ischaemic stroke.^1^ In turn, HF is associated with substantially increased mortality among AF patients.^2^ Nevertheless, while international guidelines stress the importance of individualized clinical management,^3,4^ prevention and prediction of HF remain exceedingly challenging in AF patients.

AF is known to have a strong heritable component. Notably, large genetic association studies (GWAS) have identified hundreds of genetic variants associated with arrhythmia.^5,6^ At the same time, AF is common in heritable cardiomyopathies and may be an early indicator of such genetic cardiomyopathy,^7^ especially in a young AF patient with few comorbidities.^8^ These findings suggest that genetic factors contribute to the susceptibility of AF patients to develop HF, leaving an opportunity for genome-informed personalized medicine. Indeed, we recently showed that certain rare genetic variants increase the risk of HF in people with AF.^9^ Although these rare genetic variants are more common in AF patients than in the general population, they are still quite rare. In contrast, common genetic variants could be relevant to a larger segment of the AF population. While it is plausible that common genetic variants contribute to HF risk in AF patients, the role of such variants in HF risk stratification remains underexplored.

Hereto, Anh et al.^10^ studied the potential role of a polygenic risk score (PRS), built from common genetic variants, for the prediction of HF among AF patients. They developed the PRS from a prominent HF GWAS,^11^ and employed a statistical approach to ensure data from UK Biobank individuals did not contribute to the PRS development. Next, they applied this new PRS score to a cohort of 21 167 individuals with AF—without any prior HF diagnosis—from the UK Biobank, a well-known large prospective cohort from the United Kingdom. In this cohort, they examined if participants in the upper two tertiles of the PRS score, labelled moderate-high, had a relatively higher risk of incident HF compared with the lowest tertile, labelled low. Subsequently, they stratified the analysis by age of AF onset with a cut-off of 60 years for early-onset AF. Finally, they investigated if the PRS would improve their own clinical risk model for HF.

In their work, Anh *et al*. found that AF patients with a moderate-high PRS score had an approximately 20% higher risk of HF over a median follow-up of 3.8 years, as compared with patients with low polygenic risk. This increase in HF risk was even more pronounced when stratifying the cohort by age of diagnosis. In individuals with early-onset AF (*N* = 2231), a moderate-high PRS was associated with an over two-fold higher risk of future HF. Next, Anh *et al*. constructed a clinical risk model for HF which had a modest area under the curve (AUC) of 0.64. Once they added the PRS to their clinical risk model, correct prediction of HF showed a modest improvement in all AF patients (8.8%), but the predictive ability of the model increased remarkably in early-onset AF patients by 29.7%, AUC 0.71.

Among these early-onset AF patients, the authors then showed that PRS was most valuable for individuals without prevalent diabetes, hypertension, or a history of MI. This finding suggests an important role for PRS in prognosis, as these individuals would be classified as low-risk when assessed solely by conventional cardiometabolic risk factors. On the other hand, the PRS by Anh *et al*. did not contribute to the risk of HF in people with AF and a history of MI, indicating that the influence of this HF PRS is negligible when AF is associated with overt structural insult to the heart. As such, the extensive subgroup analyses form a significant strength of the paper, showing which patients would potentially benefit from PRS-based stratification. We do note, however, that subgroups were individually small and additional replication in other cohorts will be needed before definitive statements can be made about specific subgroups within the early-onset patients. Moreover, it should be noted that the study population was restricted to individuals of European ancestry, thus the utility of PRS for HF risk prediction in the AF still needs to be validated in diverse cohorts.

That being said, the results in the early-onset AF group are striking given the recent attention to genetics in young AF patients in recent AF guidelines. The new ACC/AHA guidelines provided an initial 2B recommendation to consider genetic testing in patients with very early-onset AF (<45 years).^12^ Similarly, the 2024 ESC/EACTS guidelines also emphasize personalized management of risk factors and comorbidities in AF.^4^ Consistent with the findings by Ahn *et al*., previous work on rare genetic variation also indicated that the influence of genetics is increasingly important with younger ages of AF onset.^9,13^ Thus, it is conceivable that an HF PRS might be a meaningful addition to genetic testing for individuals with very early-onset AF, and in particular for the estimated 85–95% of patients without causative rare genetic variants.^9,14^ While we note that clinical genetic testing currently focuses on rare genetic variants and does not assess PRS, these results indicate that both genetic approaches could add to the prognosis of early-onset AF patients, in the future. Nevertheless, we stress again that additional studies of young AF patients—assessing both common and rare genetic variation^9^—are needed.

The meaningful contribution of PRS to HF prediction—especially among young AF patients with few comorbidities—also provides a compelling rationale for future research integrating both genetic and dynamic molecular approaches, like proteomics and metabolomics, to elucidate the distinct pathways driving HF development in this patient population.^15^ This is especially important for therapeutic strategies in these young patients, as current interventions, like lifestyle modifications, cardiovascular risk management, and increased clinical surveillance, would likely not reach their full potential in patients with few comorbidities. In addition to improving mechanistic and therapeutic understanding of AF and HF, future studies could also assess whether circulating proteins or metabolites, potentially paired with imaging data, might further improve HF prediction models on top of clinical and genetic risk.^16^

Overall, a more holistic approach to HF prediction in the AF population is needed. The study by Anh *et al*. identifies a significant genetic component to HF risk in AF patients, which calls for increased efforts to unravel how rare and common variants leave the fibrillating heart more vulnerable to HF. More information on how rare and common genetic variants contribute to the development of HF could open the door to personalized screening tools and targeted therapies to prevent, detect, or delay the onset of HF in (early-onset) AF patients.

## Data Availability

No datasets were utilized for this work.
